# Influence of nanosilicon on drought tolerance in plants: An overview

**DOI:** 10.3389/fpls.2022.1014816

**Published:** 2022-12-01

**Authors:** Krishan K. Verma, Xiu-Peng Song, Munna Singh, Hai-Rong Huang, Rajan Bhatt, Lin Xu, Vinod Kumar, Yang-Rui Li

**Affiliations:** ^1^ Key Laboratory of Sugarcane Biotechnology and Genetic Improvement (Guangxi), Ministry of Agriculture and Rural Affairs/Guangxi Key Laboratory of Sugarcane Genetic Improvement/Sugarcane Research Institute, Guangxi Academy of Agricultural Sciences, Nanning, Guangxi, China; ^2^ Department of Botany, University of Lucknow, Lucknow, India; ^3^ Punjab Agricultural University, Regional Research Station, Kapurthala, Punjab, India; ^4^ Department of Botany, Government Degree College, Ramban, India

**Keywords:** antioxidants, reactive oxygen species, water scarcity, nanosilicon, stress resistance efficiency, plants

## Abstract

Insufficient availability of water is a major global challenge that plants face and that can cause substantial losses in plant productivity and quality, followed by complete crop failure. Thus, it becomes imperative to improve crop cultivation/production in unsuitable agricultural fields and integrate modern agri-techniques and nanoparticles (NPs)-based approaches to extend appropriate aid to plants to handle adverse environmental variables. Nowadays, NPs are commonly used with biological systems because of their specific physicochemical characteristics, viz., size/dimension, density, and surface properties. The foliar/soil application of nanosilicon (nSi) has been shown to have a positive impact on plants through the regulation of physiological and biochemical responses and the synthesis of specific metabolites. Reactive oxygen species (ROS) are produced in plants in response to drought/water scarcity, which may enhance the ability for adaptation in plants/crops to withstand adverse surroundings. The functions of ROS influenced by nSi and water stress have been assessed widely. However, detailed information about their association with plants and stress is yet to be explored. Our review presents an update on recent developments regarding nSi and water stress in combination with ROS accumulation for sustainable agriculture and an eco-friendly environment.

## Introduction

Insufficient water availability is a big problem that causes substantial global losses in plant performance and productivity both quantitatively and qualitatively. The plants are the main producers of bio-ecosystems, and they often have to respond to different agroclimatic conditions such as water deficits, submergence/soil flooding, heavy metal toxicity, pesticides/herbicides, salt stress, high and low light intensities, pests/insects, and pathogens (Ghorbanpour et al., 2020; Verma et al., 2022a; Verma et al., 2022b). It is well documented that water deficits may impair photosynthetic and metabolic processes associated with the regulation of proper plant growth and development (Verma et al., 2021).

Enhancing plant yield in marginal agricultural areas is an integral part of the second agro-technological revolution, and NP-based approaches have already demonstrated the significance of their added advantages in plants: the ability to adapt to harsh atmospheric conditions (Adisa et al., 2019). Consequently, research into nanomaterials (NMs) has received significant attention due to their unique physical-chemical characteristics. Furthermore, in terms of diffusivity, electrical resistivity, and electrical conducting characteristics, nanoparticles are considered completely different from bulk materials (Khashan et al., 2016; Mary et al., 2019). Upon comparison with their respective bulk forms, nanoparticles are found to be almost “identical” with molecular sizes of around 1–100 nm in diameter with specific characteristics (Verma et al., 2022a). The rapid use and accumulation of engineered nanoparticles (ENPs) in the environment, and their unknown interactions with various species, revealed SNPs that are more toxic, which has raised concerns regarding environmental health (Nel et al., 2006; Thwala et al., 2016). Apart from this, the use of several ENPs has had a negative impact on the natural environment, including the quality of the water, air, and soil (Hashimoto et al., 2017). Plants are crucial components of the biosphere and actively interact with ENPs, and ENPs could thus easily be absorbed by plant roots, enter the food chain through dietary intake, and eventually have an adverse impact on human health (Pittol et al., 2017).

Silicon (Si) is a potential element that may help plants when responding to a water deficit by providing structural cellular stability, including for cell organelles. Silicon constitutes a major part of the soil in the form of silicate and aluminum silicates. It exists as monomeric or monosilicic acid in the soil solution, where it may be taken by plant roots and supplied to above-ground plant parts (Verma et al., 2020; Song et al., 2021). Silicon nanoparticles (nSi) demonstrated a potential role in enhancing proper plant development, especially crop productivity during biotic or abiotic stresses (Adisa et al., 2019; Hussain et al., 2019; Rajput et al., 2021). The studies made so far observed the potential influence of nSi through foliar/soil application or seed priming to acquire abilities to combat metal toxicity, UV-B radiation, alkalinity, salinity, water deficit or water surplus, low or high light intensity, and oxidative stress (Siddiqui et al., 2014; Cui et al., 2017; Hussain et al., 2019; Elsheery et al., 2020; Rajput et al., 2021).

**Figure 1:**
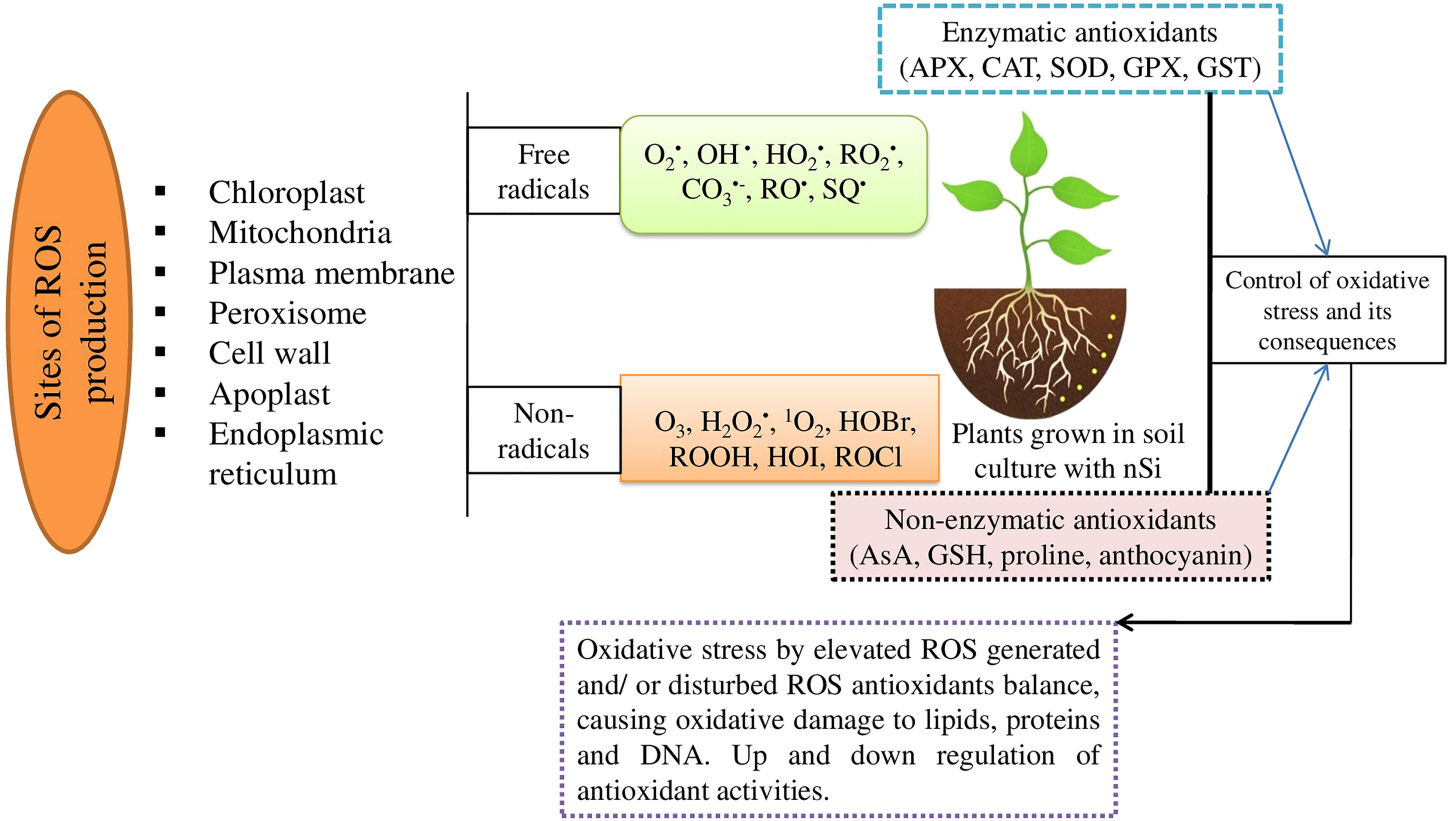
Schematic presentation indicating the possible causes for overproduction of ROS that could damage the normal functions of the cells. ${\text{O}}_{2}^{\cdot }$ , Superoxide; OH^·^, hydroxyl; ${\text{HO}}_{2}^{\cdot }$ , hydroperoxyl; ${\text{RO}}_{2}^{\cdot }$ , peroxyle; ${\text{CO}}_{3}^{\cdot -}$ , carbonate; RO^·^, alkoxyl; SQ^·^, semiquinone; O_3_, ozone; H_2_O_2_, hydrogen peroxide; ^1^O_2_, singlet oxygen; HOBr, hypobromous acid; ROOH, hydroperoxides; HOI, hypoiodous acid; and HOCl, hypochlorous acid.

Insufficient water may promote the generation of ROS: hydrogen peroxide (H_2_O_2_), hydroxyl radical (^•^OH), and superoxide anion ${\text{(O}}_{2}^{\cdot })$ . Plants have developed a number of adaptative and defensive mechanisms, including the activation of efficient enzymatic and non-enzymatic antioxidative defense systems, to ameliorate the damaging effects of ROS (Adisa et al., 2019; Verma et al., 2022a) (**Figure 1**); limited findings have been made regarding the effects of nSi on the mechanisms of ROS during the water-deficit condition. Therefore, various possibilities are reported in our review on the importance of nSi in regulation of ROS mechanisms linked with stress tolerance in plants and crop production for sustainable agriculture.

## Reactive oxygen species in stress responses

The generation of ROS is found to be triggered from available molecular oxygen *in vivo*. The adaptive mechanism of ROS detoxification in plant cells is also supported by a variety of adaptive metabolic strategies that balance the level of free transient metals (Fe^2+^) and downregulate the production of ROS to prevent the formation of an excessive amount of hydroxyl radicals *via* the Fenton reaction (Choudhury et al., 2017). All cellular compartments (the apoplast, chloroplast, peroxisome, mitochondria, vacuole, cytosol, and nuclei) continuously produce ROS, and the ROS gene network regulates the process (Choudhury et al., 2017). The plants respond to environmental challenges in controlling the formation of ROS (Foyer and Noctor, 2016) in the form of the singlet oxygen (^1^O_2_), superoxide anion ${\text{(O}}_{2}^{-})$ , hydroxyl radical (^•^OH), and hydrogen peroxide (H_2_O_2_), all of which contain oxygen and are extremely reactive due to their electron receptivity. ROS production also results from aerobic metabolic activities like photosynthesis and enzymatic and non-enzymatic processes (Apel and Hirt, 2004; Jiang et al., 2021).

The stresses may enhance ROS formation by raising ROS levels in plants (Ahmad et al., 2010). Besides these events, genetically programmed enzymatic mechanisms such ${\text{O}}_{2}^{-\cdot }$ generation by NADPH oxidases or the production of photoactivation phytoalexins may actively produce ROS in response to stress (Flors and Nonell, 2006). By reprogramming gene expression, altering cell walls, and rarely inducing programmed cell death (i.e., the hypersensitive response) to protect against viruses and other hazards, ROS may help cells adapt to stress (Waszczak et al., 2018). However, if the plant’s antioxidant system is unable to regulate the timing and amount of ROS generation, ROS may disrupt the plants’ own membrane lipids, proteins, and DNA (Demidchik, 2015; Czarnocka and Karpinski, 2018). Apart from this, a few studies also indicated the time and intensity of ROS generation and their types, produced in various cellular compartments to mitigate the consequences of oxidative stress (Gadjev et al., 2006; Shapiguzov et al., 2012).

## Defense mechanism against ROS generation

ROS has been confirmed to play an important role in connecting various morpho-physiological processes in living organisms (Jiang et al., 2021). The chloroplast, mitochondria, peroxisome, and apoplast are the four important ROS-producing organelles (Jiang et al., 2021) subjected to environmental stresses. The production of ROS may also maintain balance in the energy transfer between PSII and PSI under stressed conditions (Kleine and Leister, 2016). The alleviation of chloroplastic ROS was found to be influenced by an array of ROS-scavenging enzymes and Fe^-^ and CuZn-SODs, Asada-Foyer-Halliwell pathways, and excess levels of antioxidatives (Choudhury et al., 2017). ROS may cause proteins to undergo reversible and/or irreversible modifications and may also alter plant metabolism through transcriptional regulatory systems along with sulfonylation, carbonylation, glutathionylation, and S-nitrosylation found to be regulated by ROS-induced post-translational modifications (Choudhury et al., 2017).

Various reports demonstrated the efficiency of ENPs in agriculture (Manjunatha et al., 2016; Adisa et al., 2019; Verma et al., 2022a; Verma et al., 2022b). However, the majority of earlier investigations on the interactions between plants and ENPs concentrated on the possible toxicity of nanoparticles to higher plants. ENPs have been found to have both a significant and insignificant impact on plants (Hatami and Ghorbanpour, 2014). Generally, the formation of ROS in plant cells creates an interface for the phytotoxicity of ENPs (Melegari et al., 2013). According to Rico et al. (2015) and Ma et al. (2015), plants typically produce ROS as a byproduct of metabolic processes in chloroplasts and other organelles. However, excessive ROS production may harm the photosynthetic apparatus and other physiological and biochemical systems, eventually leading to the activation of defense mechanisms in plants, like increased antioxidant activity (Du et al., 2017). Additionally, the formation of ENPs may activate defense systems by activating antioxidant enzymes to eliminate the toxicity of ROS (Rai et al., 2018). The variations in nSi types, exposure situations, and variety of crops may influence its generation/accumulation levels and antioxidant responses (Ghorbanpour et al., 2020).

## Nanosilicon and ROS action of mechanism in response to water deficits

Reactive oxygen species are generally formed as a by-product of plant metabolic processes. Numerous ecological conditions may cause overproduction of ROS in plants with progressive oxidative damage (Chahardoli et al., 2020; Elsheery et al., 2020). The activities of antioxidant enzymes in plants increase in response to atmospheric environmental variables (Adisa et al., 2019). Plant tolerance to oxidative stress may be improved by increased antioxidant enzyme activities (Mittler, 2002) with the activation to catalase (CAT), a crucial enzyme that scavenges ROS in plant cells. CAT participates in the main defensive mechanism against the enhancement of H_2_O_2_ and may control H_2_O_2_ levels in cells (Song et al., 2016). The enhanced CAT may be attributed to more elevated superoxide dismutase (SOD) and higher production of H_2_O_2_ (Chahardoli et al., 2020).

Peroxidase (POD) influences the production of lignin, ethylene, and the breakdown of indole-3-acetic acid (IAA) in addition to lowering H_2_O_2_ generation under oxidative stress; it is also associated with plant tolerance to pathogens and an aid for wound healing (Song et al., 2016). By accelerating the dismutation of free hydroxyl radicals to H_2_O_2_ and O_2_, SOD was found to be essential (Chahardoli et al., 2020), enhancing the development of plant incase associated with nano-based approaches. Thus, a variety of nano-sized particles have been manufactured recently to enhance the productivity of crops in marginal agricultural areas subjected to adverse environmental variables (Elsheery et al., 2020; Rajput et al., 2021; Verma et al., 2022a).

Plants need to balance/maintain their ROS levels inside the cell to deal with oxidative damage during environmental stress, and this is accomplished through complex enzymatic activities, such as SOD, CAT, POD, GR, and APX, and also non-enzymatic activities, i.e., carotenoids, non-protein amino acids and phenolic compounds (Yang et al., 2010; Adisa et al., 2019). The minimal oxidative damage indices that followed rehydrating plants with nSi indicated its major role in scavenging excessive ROS and activating antioxidant defense mechanisms in plants during drought. The excess level of ROS could be due to decreased CAT activity during unfavorable environmental variables (Chahardoli et al., 2020; Verma et al., 2022a; Verma et al., 2022b). The enhanced CAT activity in the plants associated with nSi reveals an enhancement in the ROS-scavenging capacity of stressed plants, which is accompanied by plant protection/production from oxidative damage. POD activity was substantially enhanced during water-stressed conditions; the production of H_2_O_2_ found apparently beyond capacity of plant cells creates ROS formation/oxidative stress in plants. POD facilitates the conversion of H_2_O_2_ into OH˙ (Chen and Schopfer, 1999). Plant cells are protected against oxidative stress and lipid peroxidation, APX—a component of the ascorbate-glutathione (AsA-GSH) cycle, and the major ROS scavenging process (Candan and Tarhan, 2003; Elsheery et al., 2020). Hence, activation of SOD, if accompanied by the other ROS-scavenging enzymes, enables defense strategies to alleviate oxidative burst during times of drought in plants (Ghorbanpour et al., 2013) (**Figure 1**).

## Conclusions and future prospects

To alleviate the various adverse environmental variables, recent agricultural approaches need to fine-tune the intrinsic capabilities of cellular systems/plants. Agricultural applications and approaches may also aim to enhance plant production during climate change, which may cause major losses to overall plant performance and productivity. Hence, we require the implementation of newer approaches to ensure food security in developing countries, adapting to cropping systems growing under changing environmental conditions associated with application of suitable irrigation methods and fertilizers. To solve this concern, effective low-cost agro-technologies will be useful for agrofarmers. ROS appear to be important to plants faced with environmental challenges, as ROS enable them to change their metabolic activities and develop a suitable acclimation response: as long as the cells are balanced, there are adequate energy stores to detoxify ROS. nSi has been found to have a significant role in agro-ecosystems and to increase stress resistance capacities for future sustainable agriculture.

## Author contributions

KV and Y-RL conceived the article. All authors contributed to the discussion and approved the final manuscript for publication.

## Funding

This research was financially supported by the Guangxi Innovation Teams of Modern Agriculture Technology (nycytxgxcxtd-2021-03), Youth Program of National Natural Science Foundation of China (31901594), The National Natural Science Foundation of China (31760415), Guangxi Natural Science Foundation (2021GXNSFAA220022), Fund of Guangxi Academy of Agricultural Sciences (2021YT011) and Guangxi Key Laboratory of Sugarcane Genetic Improvement Project (21-238-16-K-04-02).

## Acknowledgments

The authors would like to thank the Guangxi Academy of Agricultural Sciences, Nanning, Guangxi, China, for providing the necessary facilities for this study.

## Conflict of interest

The authors declare that the research was conducted in the absence of any commercial or financial relationships that could be construed as a potential conflict of interest.

## Publisher’s note

All claims expressed in this article are solely those of the authors and do not necessarily represent those of their affiliated organizations, or those of the publisher, the editors and the reviewers. Any product that may be evaluated in this article, or claim that may be made by its manufacturer, is not guaranteed or endorsed by the publisher.
